# Increased Expression of Chitinase 3-Like 1 in Aorta of Patients with Atherosclerosis and Suppression of Atherosclerosis in Apolipoprotein E-Knockout Mice by Chitinase 3-Like 1 Gene Silencing

**DOI:** 10.1155/2014/905463

**Published:** 2014-03-04

**Authors:** Zushun Gong, Shanshan Xing, Fei Zheng, Qichong Xing

**Affiliations:** ^1^Department of Cardiology, Qianfoshan Hospital, Shandong University, 16766 Jingshi Road, Jinan 250014, China; ^2^Department of Cardiology, Shandong University of Traditional Chinese Medicine, Jinan 250355, China

## Abstract

*Introduction*. The purpose of this study was to investigate the changes of chitinase 3-like 1 (CHI3L1) in the aorta of patients with coronary atherosclerosis and to determine whether inhibition of CHI3L1 by lentivirus-mediated RNA interference could stabilize atherosclerotic plaques in apolipoprotein E-knockout (ApoE^−/−^) mice. *Methods*. We collected discarded aortic specimens from patients undergoing coronary artery bypass graft surgery and renal arterial tissues from kidney donors. A lentivirus carrying small interfering RNA targeting the expression of CHI3L1 was constructed. Fifty ApoE^−/−^ mice were divided into control group and CHI3L1 gene silenced group. A constrictive collar was placed around carotid artery to induce plaques formation. Then lentivirus was transfected into carotid plaques. *Results*. We found that CHI3L1 was overexpressed in aorta of patients with atherosclerosis and its expression was correlated with the atherosclerotic risk factors. After lentivirus transduction, mRNA and protein expression of CHI3L1 were attenuated in carotid plaques, leading to reduced plaque content of lipids and macrophages, and increased plaque content of collagen and smooth muscle cells. Moreover, CHI3L1 gene silencing downregulated the expression of local proinflammatory mediators. *Conclusions*. CHI3L1 is overexpressed in aorta from patients with atherosclerosis and the lentivirus-mediated CHI3L1 gene silencing could represent a new strategy to inhibit plaques progression.

## 1. Introduction

Coronary artery disease (CAD) has become the principal cause of death in the world. As a systematic disease which usually affects large- and medium-sized elastic and muscular arteries all over the body, atherosclerosis is the underlying pathology of most CADs. Substantial evidence supports the concept that atherosclerosis is a chronic inflammatory disease characterized by the deposition of fibrous matrix and lipids in the arterial wall. According to the “response-to-injury” hypothesis, the endothelial denudation and endothelial dysfunction caused by some risk factors are the first step in the development of atherosclerosis [1]. The activated endothelial cells facilitate monocytes infiltration into the vessel wall. Then, these monocytes differentiate into macrophages, which accumulate lipids from the circulation and remain in the vessel wall, thereby becoming foam cells. These cells mentioned above can synthesize and release proinflammatory molecules such as tumor necrosis factor *α* (TNF-*α*), monocyte chemoattractant protein 1 (MCP-1), and interleukin 1 (IL-1), which can induce further accumulation of monocytes and migration and proliferation of vascular smooth muscle cells (SMCs) [2].

Chitinase 3-like 1 (CHI3L1), also called cartilage glycoprotein 39 or YKL-40 in human and breast regression protein 39 in mice, is a 40 kDa chitin-binding glycoprotein without chitinase activity, and it has been shown to act as an important regulator of acute and chronic inflammation [3, 4]. It is secreted by a variety of cells, including SMCs and macrophages and is found in tissues with inflammation and extracellular tissue remodeling. Up to now, several studies have shown an important link between CHI3L1 and inflammation or metabolic diseases, including asthma [5], hypertension [6], diabetes mellitus [7, 8], insulin resistance [9], and atherosclerosis [10, 11], and naturally believe that CHI3L1 may be a potential biomarker and therapeutic target for the related diseases.

Although the relationship between CHI3L1 and CAD is important, there is a controversy in the association between blood CHI3L1 levels and the severity of atherosclerosis. One study investigating the role of CHI3L1 in patients with peripheral arterial disease showed that severity of atherosclerosis is associated with higher blood CHI3L1 levels [12], and another paper concluded that circulating CHI3L1 was not specifically related to the size of atherosclerotic stenosis [13]. These conflicting results may be due to the differences in the study participants and diagnostic modality for evaluation of coronary artery and severity of atherosclerosis. In order to elucidate the relationship between CHI3L1 and CAD and furthermore verify the therapeutic value of CHI3L1, we designed this study. First, we investigated the correlation between CHI3L1 expression and pathogenesis of atherosclerosis by measuring the changes of CHI3L1 in the aortic tissues of patients undergoing coronary artery bypass graft (CABG) surgery. Second, we constructed lentiviral vectors, which can efficiently deliver small interfering RNAs (siRNAs) due to their stable transduction of both dividing and nondividing cells, and aimed at knocking down CHI3L1 to explore the mechanisms of CHI3L1 in atherosclerosis in apolipoprotein E-knockout (ApoE^−/−^) mice as a potential target for treatment.

We found that the expression of CHI3L1 was enhanced in aorta of patients with coronary atherosclerosis and its expression was significantly correlated with the atherosclerotic risk factors and the severity of CAD. In addition, the interference with CHI3L1 expression resulted in an improvement of atherosclerotic burden and plaque stability in ApoE^−/−^ mice.

## 2. Methods

### 2.1. Study Population

From 2011 to 2012, 39 patients with CAD scheduled for CABG surgery were investigated and were defined as a research group. After admission, a detailed disease history, physical examination, and routine laboratory tests were carried out to establish a clinical diagnosis. Special attention was paid to the atherosclerotic risk factors including status of smoking, hypertension, and diabetes mellitus. All CAD patients were confirmed by coronary angiography (CAG). CAG was obtained using 5F catheters with Judkins method. The Gensini score system was used to assess the CAD severity, according to the distribution, extent, and severity of coronary artery stenosis. The exclusion criteria were acute or chronic infection, stroke, acute, or chronic liver or kidney disease, autoimmune disease, neoplasm, and trauma. Eleven normal subjects who donated kidneys were investigated as the control group. All kidney donators were blood relatives of recipients. They did not suffer from any diseases including CAD, hypertension, diabetes mellitus, trauma, malignancies, and acute or chronic inflammatory status. In addition, they were nonsmokers and reported no long-term drug use.

Aortic specimens were obtained from the aorta that was routinely removed during CABG surgery as a button hole. In addition, the discarded renal arterial tissues without atherosclerotic lesions were collected from the 11 subjects who donated kidneys. In each group, the arterial tissues were randomly distributed for histological analysis and western blot analysis.

This study protocol conforms to the principles of the Declaration of Helsinki and was approved by the ethics committee of the Shandong Provincial Qianfoshan Hospital. All patients and normal subjects who participated in this study signed the informed consent forms.

### 2.2. Biochemical Analysis

Venous blood samples of patients in the research group and normal subjects in the control group were obtained after a 12 h fasting for measurement of serum triglycerides (TG), total cholesterol (TC), low density lipoprotein cholesterol (LDL-C), high density lipoprotein cholesterol (HDL-C), lipoprotein (a) [Lp (a)], and apolipoprotein A (ApoA), as well as apolipoprotein B (ApoB). All the items mentioned above were measured with standard laboratory techniques by the Department of Clinical Chemistry, Shandong Provincial Qianfoshan Hospital.

### 2.3. Cell Culture

The 293T human embryonic kidney cell line, which expresses simian virus 40 large T antigen and facilitates the optimal production of viruses, and the RAW264.7 mouse macrophage cell line were purchased from the Chinese Academy of Typical Culture Collection cell bank (Shanghai, China). All cells were cultured in Dulbecco's modified Eagle's medium supplemented with 10% fetal bovine serum, 100U/mL penicillin, and 100 ug/ml streptomycin. All cells were incubated in a 37°C humidified incubator with 95% air and 5% CO_2_.

### 2.4. Lentivirus Construction and Target Screening for RNAi

Four different sequences (sites A, B, C, and D) of CHI3L1 gene in mice were designed as the target for RNA interference (RNAi) (Genepharma, Shanghai, China). The sequence of site A was 5′-GCGACAACATGCTTAGCACATTTCAAGAGAATGTGCTAAGCATGTTGTCGCTT-3′; the sequence of site B was 5′-GGCCATTGACACTGGCTATGATTCAAGAGATCATAGCCAGTGTCAATGGCCTT-3′; the sequence of site C was 5′-GCACTGGATTTGGATGATTTCTTCAAGAGAGAAATCATCCAAATCCAGTGCTT-3′; the sequence of site D was 5′-GCCAGAAGGACACTAGGTTTGTTCAAGAGACAAACCTAGTGTCCTTCTGGCTT-3′. As a control, the scrambled sequence (mock siRNA) was 5′-GTTCTCCGAACGTGTCACGTTTCAAGAGAACGTGACACGTTCGGAGAACTT-3′. Then the pShuttle vectors containing the mouse CHI3L1 RNAi sequences were constructed. A lentivirus was produced by cotransduction of the siRNA expression pShuttle vectors pGag/Pol, pRev, and pVSV-G into the 293T cells. Then lentiviruses were used to transfect RAW264.7 cells. To screen the target for the most effective gene interference, RAW264.7 cells were collected for the following polymerase chain reaction (PCR) and western blot experiment at 72 h and 96 h after transduction, respectively. Nonlentivirus and lentivirus containing mock siRNA transduction served as controls.

### 2.5. Animal Experiment

We obtained 50 male ApoE^−/−^ mice, 8 weeks old, from the Beijing University Animal Research Center (Beijing, China). All mice were housed five per cage and were fed a high-fat diet (15% cocoa butter and 0.25% cholesterol) with free access to water. The mice were divided into 2 groups (*n* = 25  each): control group and CHI3L1 lentivirus silenced group. The atherosclerotic model was as previously described [14]. In brief, after anesthesia by intraperitoneal injection of pentobarbital sodium (40 mg/kg), a constrictive silica collar (inner diameter, 0.3 mm; outer diameter, 0.5 mm; and length, 2.5 mm) was placed on the right common carotid artery of mice. Eight weeks after surgery, the carotid collars were removed and the proximal right common carotid artery and the distal right internal and external carotid arteries were temporarily ligated. Then 20 *μ*L of lentiviral suspension at 1 × 10^9^ TU/mL was instilled into the right common carotid artery, left *in situ* for 15 min before closure of the skin incision [15].

All animal procedures were performed in accordance with the institutional guidelines of the Shandong Provincial Qianfoshan Hospital.

### 2.6. Tissue Preparation and Histological Analysis

Immunohistochemical analyses were performed according to routine laboratory methods [16]. For arterial tissues obtained from CABG surgery and kidney donators, the specimens were fixed in 4% buffered formalin overnight at 4°C, then dehydrated in an ascending ethanol series, routinely embedded in paraffin, and sectioned at 3 *μ*m. After conventional deparaffinage, hydration, and antigen retrieve, endogenous peroxidase was inactivated by 3% hydrogen peroxide. The sections were incubated with the rabbit antihuman CHI3L1 polyclonal antibody (diluted to 1 : 200, Bioss, Beijing, China) at 4°C for 12 h. After washings with phosphate-buffered saline (PBS), the sections were incubated with the goat anti-rabbit IgG polymer at room temperature for 30 min. Then the sections were visualized with 3,3-diaminobenzidine to produce a brown product and then counterstained with hematoxylin, dehydration, transparention, and fixation by neutral resins. As negative controls, rabbit nonimmune serum was applied parallel with rabbit anti-human CHI3L1 polyclonal antibody.

Mice were sacrificed by intraperitoneal injection of pentobarbital sodium (200 mg/kg) 4 weeks after transduction and were perfused with PBS through the left ventricle. The right common carotid artery was carefully excised and immersed in 4% formaldehyde. Six cross-sections in each mouse were used for a particular type of staining. One section was stained with hematoxylin and eosin (H&E). Another section was immunostained with rabbit anti-mouse CHI3L1 polyclonal antibody (diluted to 1 : 300, Santa Cruz, sc:98954). Collagen and lipids deposition in plaques were identified by Sirius red staining and oil red O staining, respectively. SMCs and macrophages were immunostained with anti-*α*-actin antibody (diluted 1 : 300, Boshide, Wuhan, China) and macrophage-specific antibody (diluted 1 : 200, Boshide, Wuhan, China), respectively. An automated image analysis system (Image-Pro Plus 5.0, Silver Spring, MD) was used for quantitative measurements. The positive-staining area of collagen, lipids, SMCs, and macrophages was quantified by computer-assisted color-gated measurement, and the ratio of positive-staining area to intimal area was calculated. The vulnerability index was calculated by the following formula: positive-staining area of (macrophages + lipids)/positive-staining area of (SMCs + collagen).

In addition, small part of fresh mice arterial tissues was used to undergo electron microscope examination. In brief, the arterial tissues were placed in 2.5% glutaraldehyde and 2% paraformaldehyde for 1 h; then the vessels were cut into approximately l mm × 1 mm × 1 mm and returned to the fixative for another 1 h. The tissues were postfixed in 1% osmium tetroxide for 1 h followed by staining with 2% uranyl acetate for 1 h. Then the tissues were dehydrated through ethanol and were embedded in Spon812. Finally, 50 nm sections were stained with uranyl acetate followed by lead citrate and examined in a JEM-1010 electron telescope (JEOL, Japan).

### 2.7. Quantitative Real-Time RT-PCR Analysis

The mRNA expression levels of *β*-actin and CHI3L1 in RAW264.7 cells, *β*-actin, CHI3L1, TNF-*α*, MCP-1, IL-8, and matrix metalloproteinase 9 (MMP-9) in plaque tissues were quantitatively analysed using real-time RT-PCR according to the routine methods [17]. In brief, RNA was extracted with the use of TRIzol reagent (Invitrogen) in accordance with the manufacturer's instructions. The housekeeping gene *β*-actin was quantified as an internal RNA control. The forward and reverse primers were as follows: 5′-AGGCTTTGCGGTCCTGAT-3′ and 5′-CCAGCTGGTGAAGTAGCAGA-3′ for CHI3L1; 5′-CACCACGCT CTTCTGTCTACTGAAC-3′ and 5′-CCG GACTGCGTGATGTCTAAGTACT-3′ for TNF-*α*; 5′-CAGCCAGATGCAGTTAACGC-3′ and 5′-GCCTACTCATTGGGATCAT CTTG-3′ for MCP-1; 5′-ACTGAGAGTGATTGAGAGTGGAC-3′ and 5′-AACCCTCTGCACCCAGTTTTC-3′ for IL-8; 5′-CCTGGAACTCACACGACATCTTC-3′ and 5′-TGGAAACTCACACGCCAGAA-3′ for MMP-9; 5′-CACTGTGCCCATCTACGA-3′ and 5′-GTAGTCTGTCAGGTCCCG-3′ for *β*-actin. All values obtained were normalized to mouse *β*-actin and relative expression analysis involved the 2^−ΔΔCT^ method.

### 2.8. Western Blot Analysis

The protein expression levels of CHI3L1, MAPK, AKT, GAPDH, and *β*-actin in human arterial tissues, RAW264.7 cells, and plaque tissues were assayed by western blot analysis [18]. In brief, equal amounts of protein were separated on sodium dodecyl sulfate-14% polyacrylamide gels and transferred to nitrocellulose membrane. After blocking with 5% nonfat milk, the blots were washed with PBS containing 0.1% Tween 20 and incubated with an appropriate primary antibody at 4°C for 12 h. The blots were probed with antibodies against human CHI3L1 (diluted 1 : 500, Bioss, Beijing, China), mice CHI3L1 (diluted 1 : 1000, Santa Cruz), mice p44/42 MAPK (ERK1/2) (diluted 1 : 1000, CST, 4695), phospho-ERK1/2 (diluted 1 : 1000, CST, 4370), AKT (diluted 1 : 1000, CST, 4691), phospho-AKT (diluted 1 : 1000, CST, 4060), GAPDH, or *β*-actin (diluted 1 : 500, Zhongshan, Beijing, China). Then the blots were washed with Tris-buffered saline with Tween 20 and incubated with appropriate secondary antibody conjugated to horseradish peroxidase. Blots were processed for enhanced chemifluorescence using a Pierce ECL Western blotting substrate. The housekeeping gene GAPDH or *β*-actin was quantified as an internal control.

### 2.9. Statistical Analysis

Statistical analysis was performed with Statistical Package for the Social Science (SPSS 12.0) and quantitative variables are expressed as mean ± standard deviation. After testing for normal distribution of variables, Student's *t*-test was used to analyze continuous normally distributed variables. Correlations between two variables were performed by linear correlation analysis. In general, a 2-tailed *P* < 0.05 was considered statistically significant.

## 3. Results

### 3.1. Baseline Characteristics

The baseline characteristics of the two groups are summarized in Table 1. The preoperative serums TC, LDL-C, and Lp (a) of 39 patients in research group were significantly elevated, whereas serums HDL-C and ApoA were significantly decreased, compared with the control group (*P* < 0.05). In addition, the 11 kidney donors in the control group had no history of hypertension or diabetes mellitus. Moreover, all were nonsmokers.

### 3.2. Coronary Atherosclerotic Lesions in the Research Group

CAG of 39 patients undergoing CABG showed that 19 patients were with left main coronary artery lesions, 6 patients were with two coronary arteries lesions, and 14 were patients with all three coronary arteries lesions. Significant calcification and stenosis were observed in coronary arteries of all the patients in the research group. The average Gensini score of the 39 patients was 62.25 ± 21.77.

### 3.3. Presence of CHI3L1 in the Human Arterial Tissues

**Figure 1:**
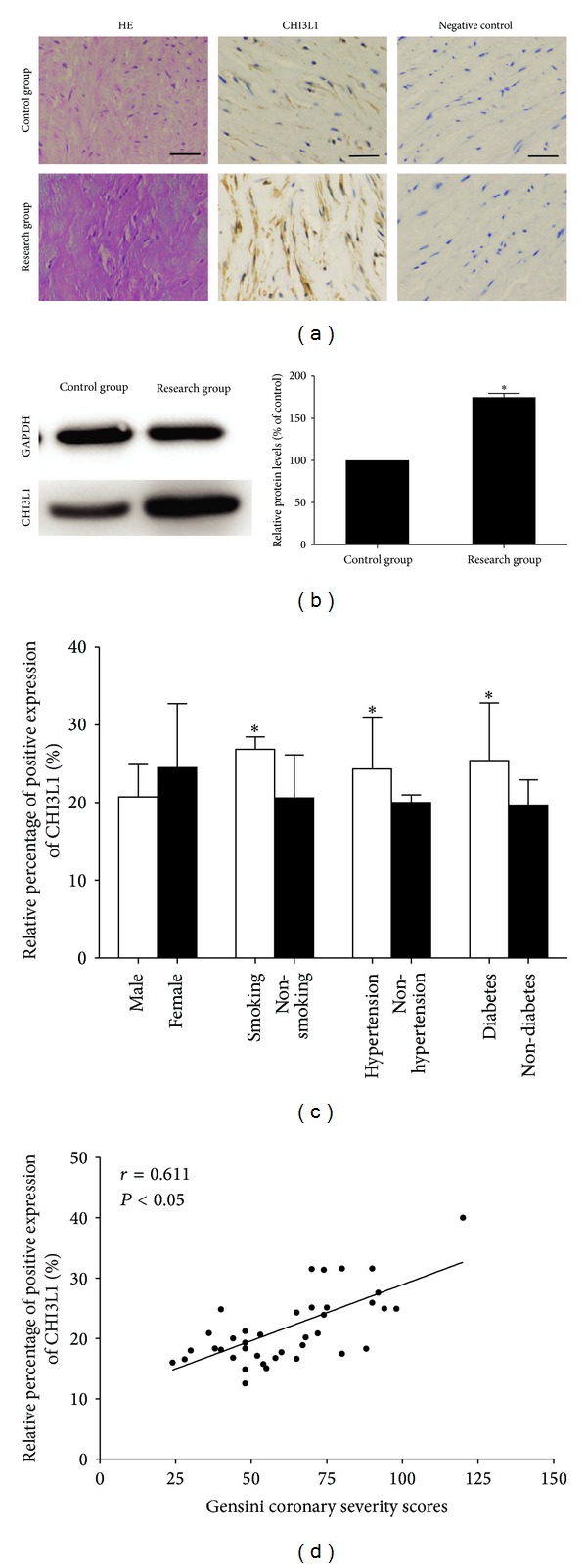
(a) Immunohistochemical staining of CHI3L1 on sections of arterial vessels in control group and research group. The expression of CHI3L1 was increased in the arterial specimens of CAD patients in research group. (scale  bars = 100 *μ*m) (b) Western blot analysis and quantification of CHI3L1 protein expression in control group and research group. The levels of CHI3L1 protein expression were higher in research group than in control group. *P < 0.05 versus control group. (c) Quantitative analysis of arterial CHI3L1 expression in research group patients according to gender, smoking, hypertension, and diabetes mellitus. The expression levels of CHI3L1 were elevated in smokers and patients with hypertension or diabetes mellitus, whereas gender had no significant effect. *P < 0.05. (d) Relationship between arterial CHI3L1 expression and coronary severity scores. The arterial CHI3L1 expression levels were significantly correlated with coronary severity Gensini scores. Each point represents one patient.

As shown in Figure 1(a), in the arterial tissues of healthy donors little CHI3L1 expression could be demonstrated according to the immunohistochemical staining. However, the expression of CHI3L1 was elevated in the arterial specimens of CAD patients. Western blot analysis was used to evaluate the expression of CHI3L1 protein. As shown in Figure 1(b), there were significant differences of CHI3L1 expression levels between control group and research group.

### 3.4. Correlation between the Arterial CHI3L1 Expression and the Clinical Criteria of Atherosclerosis

We investigated the correlation between the relative expression levels of CHI3L1 and clinical criteria of atherosclerosis, including gender, smoking, hypertension, and diabetes mellitus. The expression levels of CHI3L1 did not differ significantly between males and females, but they differ significantly among smokers and nonsmokers, hypertensives and nonhypertensives, and diabetics and nondiabetics. As shown in Figure 1(c), the expression levels of CHI3L1 were increased in smokers and patients with hypertension or diabetes mellitus (*P* < 0.05), whereas gender had no significant effect. As shown in Figure 1(d), the linear correlation analysis revealed that arterial CHI3L1 expression levels were significantly correlated with coronary severity Gensini scores, which ranged from 24 to 120 (*r* = 0.611, *P* < 0.05).

### 3.5. Effects of Lentiviral Transduction *In Vitro*

**Figure 2:**
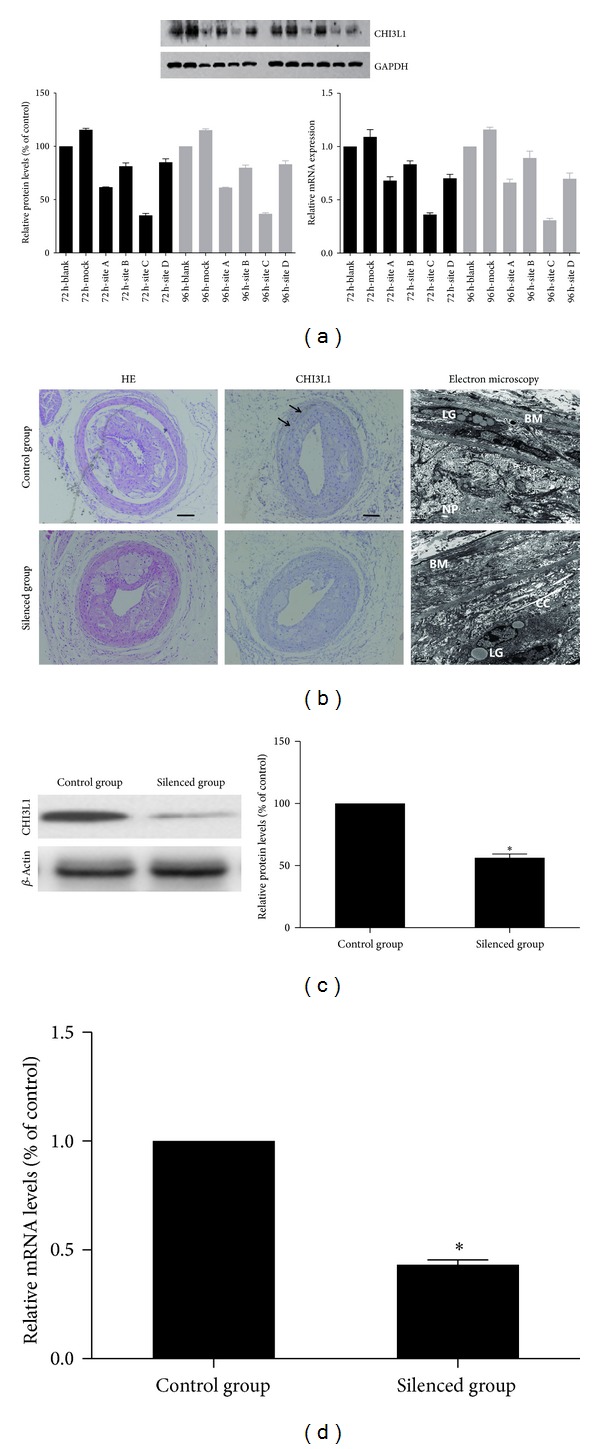
(a) Target site screening for CHI3L1 by western blot analysis and real-time RT-PCR in RAW264.7 cells. The RAW264.7 cell line was transfected with lentivirus expressing different CHI3L1 siRNAs, and gene silencing analysis showed that site C lentivirus was the most effective vector in blocking CHI3L1 expression. (b) The immunohistochemical staining of CHI3L1 and electron microscopy in control group and silenced group. In the control group CHI3L1 expression (arrow) could be demonstrated according to the immunohistochemical staining. However, little CHI3L1 was expressed in silenced group. For electron microscopy, in control group most of the endothelial cells denudated and there were a large number of lipid granules (LG) under the basement membrane (BM) in the vessel wall. The atherosclerotic plaques were occupied with necrotic particles (NP), calcification crystals (CC), and cellular debrises. However, in silenced group the number of lipid granules was relatively decreased. (scale  bars = 100 *μ*m) (c) Western blot analysis and quantification of CHI3L1 protein expression in control group and silenced group. The levels of CHI3L1 protein expression were higher in control group than in silenced group. (d) Real-time RT-PCR quantification of CHI3L1 mRNA expression in control group and silenced group. *P < 0.05 versus control group.

The RAW264.7 cell line was transfected with lentivirus expressing different CHI3L1 siRNAs, and gene silencing analysis showed that site C lentivirus was the most effective vector in blocking CHI3L1 expression. As shown in Figure 2(a), CHI3L1 knockdown clones A, B, C, and D exhibited 38, 18, 64, and 14% reduction, respectively, in protein expression and 32, 17, 65, and 30% reduction, respectively, in mRNA expression. Then site C lentivirus and mock lentivirus were selected and produced at a viral titer of 1 × 10^9^ TU/mL (Genepharma, Shanghai, China) for further *in vivo* studies.

### 3.6. Effects of Lentiviral Transduction on CHI3L1 Expression in Plaques

To evaluate the efficacy of lentivirus-mediated gene silencing *in vivo*, the changes of CHI3L1 histology, protein, and mRNA expression in atherosclerotic plaques were measured. As shown in Figure 2(b), in the control group CHI3L1 expression could be demonstrated according to the immunohistochemical staining. However, little CHI3L1 was expressed in the silenced group. For electron microscopy, in the control group most of the endothelial cells denudated and there were a large number of lipid granules under the basement membrane in the vessel wall. The atherosclerotic plaques were occupied with necrotic particles, calcification crystals, and cellular debrises. However, in the silenced group the number of lipid granules was relatively decreased. A regenerating endothelial cell was seen partially covering the denuded surface. Collagen bundles and elastic fibers were seen on the vessel side of the endothelium. SMCs migrated into plaque tissues. Western blot analysis was used to evaluate the expression of CHI3L1 protein in plaque tissues. As shown in Figure 2(c), compared with control group, the silenced group showed a reduction in CHI3L1 protein expression of 50%. In addition, there were significant differences of CHI3L1 mRNA expression levels between control group and silenced group (Figure 2(d)).

### 3.7. Effects of Lentiviral Transduction on Plaque Composition

**Figure 3:**
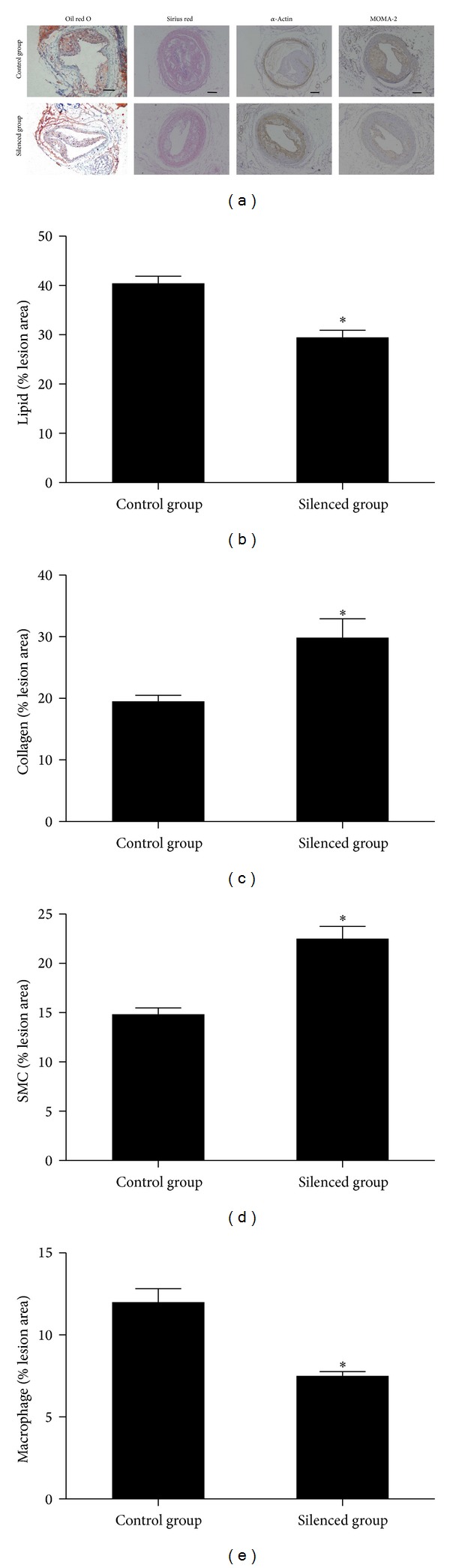
CHI3L1 gene silencing influenced plaque composition and stability. (a) Cross-sections of mice carotid arteries in the control group and silenced group were stained for lipids (oil red O), collagen (Sirius red), SMCs (*α*-actin), and macrophages (MOMA-2). The relative content of lipids (b), collagen (c), SMCs (d), and macrophages (e) in the plaque tissues. The relative contents of lipids and macrophages in plaque tissues were significantly lower in silenced group than in control group. However, the relative collagen and SMCs contents in plaque tissues were increased in silenced group than in control group. *P < 0.05 versus control group.

The relative content of lipids, collagen, SMCs, and macrophages in plaque tissues was derived by histological and immunohistochemical staining (Figure 3). The relative content of lipids in plaque tissues of the control group and the silenced group was 48.8% and 35.2%, and it was significantly lower in the silenced group than in the control group (*P* < 0.05). The relative reduction of lipids content in plaque tissues of silenced group was 27.5% as compared with control group. The relative content of collagen in plaque tissues of the control group and silenced group was 19.5% and 29.8%, and it was significantly increased in silenced group more than in control group (*P* < 0.05). The relative increase of collagen content in plaque tissues of silenced group was 53.2% as compared with control group. The relative SMCs content in plaque tissues of the control group and silenced group was 14.8% and 22.5%, and it was higher in silenced group than in control group (*P* < 0.05). The relative increase of SMCs content in plaque tissues of silenced group was 51.2% as compared with control group. The relative macrophages content in plaque tissues of the control group and silenced group was 11.9% and 7.5%, and it was decreased in silenced group more than in control group (*P* < 0.05). The relative reduction of macrophages content in plaque tissues of silenced group was 36% as compared with control group. The vulnerability index for the control group and silenced group was 1.76 ± 0.25 and 0.81 ± 0.13, and it was decreased in silenced group more than in control group (*P* < 0.05). The relative reduction in vulnerability index in silenced group was 53.8% as compared with control group.

### 3.8. Effects of CHI3L1 Gene Silencing on Inflammatory Mediators within Lesions

**Figure 4:**
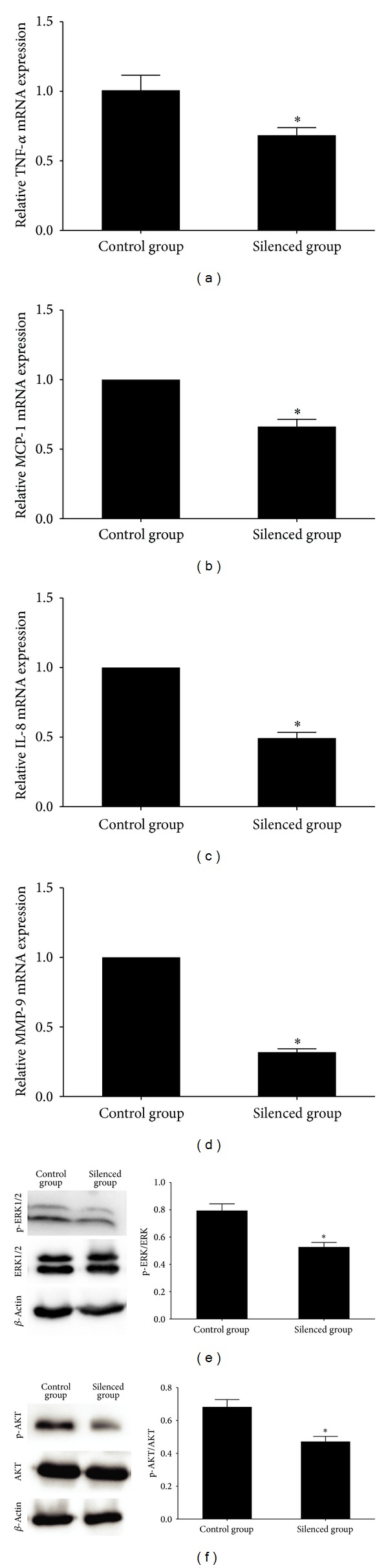
(a)–(d) Real-time RT-PCR quantification of inflammatory cytokines mRNA expression in mice carotid plaques in control group and silenced group. The silenced group showed lower mRNA expression levels of TNF-*α*, MCP-1, IL-8, and MMP-9, compared with the control group, and inhibition of CHI3L1 reduced the inductions of proinflammatory cytokines. (e)-(F) Western blot analysis and quantification of ERK1/2, phospho-ERK1/2, AKT, and phospho-AKT protein expression in control group and silenced group. The protein expression levels of phospho-ERK1/2 and phospho-AKT were decreased in silenced group compared with that in control group. *P < 0.05 versus control group.

To elucidate the molecular mechanisms by which CHI3L1 gene silencing inhibits plaques progression and stabilizes the plaques, the mRNA expression changes of the proinflammatory molecules including TNF-*α*, MCP-1, IL-8, and MMP-9 were investigated in the mice carotid arteries. As shown in Figure 4, the silenced group showed lower mRNA expression levels of TNF-*α*, MCP-1, IL-8, and MMP-9, compared with the control group (*P* < 0.05), and inhibition of CHI3L1 reduced the inductions of proinflammatory cytokines. In addition, we studied the protein expression levels of ERK1/2, phospho-ERK1/2, AKT, and phospho-AKT in the mice carotid arterial tissues and found that the protein expression levels of phospho-ERK1/2 and phospho-AKT were decreased in the silenced group compared with that in the control group.

## 4. Discussion

In the present study we found that the expression of CHI3L1 was augmented in aorta of patients with coronary atherosclerosis and its expression was significantly correlated with the atherosclerotic risk factors and the severity of CAD as quantified by coronary angiograph. More importantly, the interference of CHI3L1 resulted in an improvement of atherosclerotic burden and plaque stability in ApoE^−/−^ mice.

Inflammation and endothelial dysfunction are thought to be key processes in the progression of atherosclerosis. Several proinflammatory cytokines, acute phase-reactants, and cell adhesion molecules seem to play an important role in the development of low grade inflammation, and there is substantial evidence supporting the role of TNF-*α*, IL-6, and MCP-1 as cardiovascular risk factors and participants in the pathogenesis of atherosclerosis [1, 14, 16].

As a member of the chitinase-like proteins, CHI3L1 is highly conserved and is produced by a variety of cells such as macrophages, neutrophils, SMCs, cancer cells, and arthritic chondrocytes. Although mammals are not able to synthesize or metabolize chitin and the exact function of CHI3L1 remains unknown, recent studies have implicated CHI3L1 in different biological processes such as inflammation, tissue remodeling, fibrosis, and angiogenesis [19].

Using human arterial tissues, Boot et al. studied the CHI3L1 mRNA expression in the atherosclerotic plaques [20]. Similar to their research, we studied the CHI3L1 protein expression in the aorta of patients with coronary atherosclerosis. More importantly, to identify the potential role of CHI3L1 in atherogenesis, we investigated the relationship between CHI3L1 and cardiovascular risk factors. Gender, smoking, hypertension, diabetes mellitus, and dyslipidemia are all risk factors that contribute to the development of atherosclerosis and some evidence indicates that circulating levels of CHI3L1 have relationships with smoking [5], hypertension [6], and diabetes mellitus [7, 8]. Our results showed that aortic CHI3L1 expression had a positive correlation with smoking, hypertension, and diabetes mellitus. These results indicate that the main cardiovascular risk factors may promote the expression of CHI3L1 and CHI3L1 can influence atherosclerosis by regulating these risk factors. The Gensini scoring system is a useful tool to estimate the severity of CAD based on CAG findings. We found that there was a significant correlation between aortic CHI3L1 expression and coronary artery severity suggesting the important role of CHI3L1 in angiogenesis and in the process of atherosclerotic plaque formation.

RNA interference is an effective method for silencing mRNA, and it has been used in the treatment of several diseases [21, 22]. The use of siRNAs is more efficient than other gene-specific targeting approaches. As one kind of viral vectors, adenovirus is commonly applied in the RNA interference. However, it can lead to a marked immunogenic response, limiting associated gene expression. The lentivirus has several advantages than adenovirus, such as the high efficiency of gene transduction, long-term infection due to gene integration into the chromosome of host cells, and the absence of toxicity or immune response. Thus, lentiviral vectors expressing siRNAs was applied in our study.

Some phenotypic characteristics of atherosclerotic plaques, such as fibrous cap thickness, collagen content, and macrophage number have been widely used as indicators of plaque stability. Plaques with a thin fibrous cap and a large lipid core are considered vulnerable. In addition, in the process of acute coronary syndrome, the plaque component is more important than the plaque size. We investigated the effect of CHI3L1 gene silencing on advanced atherosclerotic lesions and found that CHI3L1 was important in the development of atherosclerotic plaques, as the CHI3L1 gene silenced group consistently showed a decreased content of macrophages and lipids and an increased content of collagen and SMCs. The morphological changes mentioned above led to a decreased plaque vulnerability index in the CHI3L1 gene silenced group compared to the control group, indicating that the plaque rupture is not apt to occur.

Studies have demonstrated that increased expression of CHI3L1 in the human atherosclerotic lesions is associated with production and activation of inflammatory factors [11, 20]. Although the membrane receptor specific for CHI3L1 binding has not been identified, the heparin-binding affinity of CHI3L1 appears to be essential for its activity, resembling the heparin-binding property of vascular endothelial growth factor. CHI3L1 can initiate MAPK and phosphoinoside-3 kinase (PI3K) by phosphorylation of the ERK1/2 and AKT, respectively, and thereby mediate signalling cascades [17, 18, 23, 24]. Both pathways have well-established roles in the propagation of mitogenic signals and play an important role in the process of atherosclerotic plaque formation. In our study, we found that the protein expression levels of phospho-ERK1/2 and phospho-AKT were decreased in silenced group compared with that in control group. As the major proinflammatory factors, TNF-*α*, MCP-1, IL-8, and MMP-9 have been detected in the human atherosclerotic lesions [25–27]. TNF-*α*, MCP-1, and IL-8 play important roles in the recruitment and activation of monocytes, macrophages, and SMCs. In turn, activated macrophages are involved in the secretion of inflammatory factors such as MCP-1. MMP-9 in the atherosclerotic plaques can degrade extracellular matrix, therefore contributing to thinning of the fibrous cap of plaques. In the present study, the mRNA expression levels of TNF-*α*, MCP-1, IL-8, and MMP-9 were decreased in silenced group compared with that in control group. These results suggested that a reduced level of these cytokines induced by interference of CHI3L1 contributed to the stabilization of atherosclerotic plaques.

## 5. Limitations

It should be mentioned that this study included a relatively small number of patients, normal subjects, and ApoE^−/−^ mice, limiting the statistical power. Although we used the Gensini scoring system to estimate the severity of coronary atherosclerosis, this score is determined using CAG and does not reflect the actual volume of atherosclerotic plaque. Thus, other diagnostic methods, such as intravascular ultrasound and angioscopy, may be needed to evaluate plaque volume. Another major limitation in the human arterial tissues study is the normal subjects, considering that healthy human aortic tissues can rarely be ethically obtained. Consequently, we selected discarded arteries from kidney donors as the control group. All kidney donors were blood relatives of the recipients. To our knowledge, up to now there are no data to show any difference in CHI3L1 expression between human ascending aorta and renal arteries. It should also be mentioned that collar-induced carotid atherosclerosis is different from natural aortic atherosclerosis in the ApoE^−/−^ mice. The carotid lesions occur in a region proximal to the constrictive collar with a prominent plaque burden, whereas the aortic lesions develop in the ascending aorta with scattered plaques of small size. Nonetheless, the carotid lesions in ApoE^−/−^ mice resemble advanced human atherosclerosis and represent the reproducible and reliable model for studies of vulnerable plaques. Finally, the local lentivirus carrying small interfering RNA can be absorbed into the circulation and cause the general effect.

## 6. Conclusions

In conclusion, the expression of CHI3L1 was enhanced in the aorta of patients with coronary atherosclerosis and its expression was significantly correlated with the atherosclerotic risk factors and the severity of CAD. More importantly, silencing of CHI3L1 diminished atherosclerotic burden and increased plaque stability in ApoE^−/−^ mice, and it might provide a new therapeutic approach to the treatment of atherosclerosis.

## Figures and Tables

**Table 1:** Baseline characteristics and serum lipid levels of subjects and patients in the two groups.

	Control group (*n* = 11)	Research group (*n* = 39)	*P*
Age, yrs	57 ± 5	60 ± 6	0.144
Male sex, *n* (%)	7 (64%)	27 (69%)	0.745
Smoker, *n* (%)	0	8 (20%)	
Hypertension, *n* (%)	0	17 (44%)	
Diabetes, *n* (%)	0	15 (38%)	
TG (mmol/L)	1.31 ± 0.46	1.95 ± 1.16	0.078
TC (mmol/L)	3.55 ± 0.26	4.99 ± 1.47	<0.01
LDL-C (mmol/L)	2.05 ± 0.21	3.15 ± 1.24	<0.01
HDL-C (mmol/L)	1.78 ± 0.25	1.26 ± 0.36	<0.01
Lp (a) (mg/dL)	19.87 ± 7.31	30.45 ± 23.53	0.025
ApoA (g/L)	1.42 ± 0.18	1.08 ± 0.25	<0.01
ApoB (g/L)	0.85 ± 0.13	0.91 ± 0.28	0.245
